# Computational Assessment of Oxygen Availability and Shear Stress in Microfluidic Cell Culture Chambers for Optimized Cell Adhesion

**DOI:** 10.3390/bios16080414

**Published:** 2026-07-31

**Authors:** Mahdi Poursaberi, Guillermo Hauke, S. Jamaleddin Mousavi, Mohamed H. Doweidar

**Affiliations:** 1 School of Engineering and Architecture (EINA), University of Zaragoza, 50018 Zaragoza, Spain; mpoursaberi@unizar.es (M.P.); ghauke@unizar.es (G.H.); 2 Aragon Institute of Engineering Research (I3A), University of Zaragoza, 50018 Zaragoza, Spain; 3 Biomedical Research Networking Center in Bioengineering, Biomaterials and Nanomedicine (CIBER-BBN), 28029 Madrid, Spain; 4 Fluid Mechanics Department, University of Zaragoza, 50018 Zaragoza, Spain; 5 Medtronic, 01600 Trevoux, France; jam.mousavi@gmail.com

**Keywords:** microfluidic cell culture, computational modeling, oxygen transport, oxygen depletion, wall shear stress, microfluidic design, SU-8 microchips

## Abstract

Microfluidic cell culture systems provide controlled microscale environments for biomedical research; however, cell viability within closed microchambers depends on adequate oxygen availability during the adhesion phase and on the mechanical stresses generated after perfusion begins. Experimental characterization of oxygen depletion and local shear stress remains challenging due to the small dimensions involved and the complexity of transport phenomena. In this study, a computational framework was developed to assess oxygen transport and hydrodynamic shear stress in an SU-8-based microfluidic culture chamber. Oxygen diffusion and cellular consumption were first modeled under static conditions to determine cell survival time prior to perfusion. The influence of chamber height on oxygen availability was investigated, and an empirical correlation was derived to predict oxygen concentration as a function of chamber geometry. Subsequently, medium perfusion was introduced, and the resulting wall shear stresses acting on adhered cells were evaluated under different flow conditions. The simulations demonstrated that chamber height significantly affects oxygen depletion time, while both chamber geometry and flow rate influence the magnitude of wall shear stress. The proposed framework provides practical design guidelines for optimizing microfluidic culture systems, enabling adequate oxygen supply and physiologically compatible mechanical conditions while reducing reliance on extensive experimental testing.

## 1. Introduction

Microfluidic cell culture systems have emerged as powerful tools for biomedical research due to their ability to reproduce physiologically relevant microscale environments while providing precise control over biochemical and mechanical stimuli. These platforms are increasingly employed in tissue engineering, drug screening, disease modeling, and organ-on-chip applications, where accurate regulation of the cellular microenvironment is essential to maintain physiological cell behavior [1,2,3]. This regulation is highly critical because cell adaptation and migration typically depend on multiple microenvironmental signaling cues [4,5].

Conventional cell culture methods, such as Petri dishes and well plates, remain widely used because of their simplicity and low cost. However, these static systems provide limited control over mass transport and mechanical stimulation, often resulting in non-uniform nutrient distribution, accumulation of metabolic waste, and poor representation of *in vivo* conditions. In contrast, microfluidic devices enable precise control of fluid flow, concentration gradients, and oxygen transport while requiring only small volumes of reagents and biological samples [6,7]. These dynamic conditions within microfluidic channels influence cell attachment, proliferation, and extracellular matrix synthesis, making computational optimization an important step before device fabrication [8,9].

These advantages make microfluidic technology particularly attractive for studying cell–microenvironment interactions under controlled conditions.

Polydimethylsiloxane (PDMS) is the material most commonly used for microfluidic fabrication owing to its low cost, optical transparency, ease of processing, and high gas permeability [10,11]. Despite these advantages, PDMS presents several limitations, including limited mechanical rigidity, hydrophobic surface properties, absorption of small hydrophobic molecules, and water evaporation through the polymer matrix [12,13]. Alternative materials such as SU-8 photoresist offer high mechanical strength, excellent optical transparency, chemical stability, and the ability to fabricate complex high-aspect-ratio microstructures [14,15,16].

However, unlike PDMS, SU-8 is essentially impermeable to gases, making oxygen transport a critical design challenge for cell culture applications [17]. While gas impermeability introduces clear mass transport challenges, rigid polymeric matrices like SU-8 remain highly advantageous for advanced device architectures, such as quasi-three-dimensional cell-based biosensors where structural fidelity is paramount [16]. However, the translation of these materials into specific biosensor or organ-on-chip device types requires a clear understanding of how microchamber geometry dictates the local microenvironment, as suboptimal scaling can directly compromise cell survival and device stability.

This limitation becomes particularly important during the initial cell seeding and adhesion stage. Before medium flow is established, cells must remain viable for a sufficient period to adhere to the microfluidic chip wall while relying exclusively on the oxygen initially dissolved in the culture medium. Consequently, chamber geometry, cellular oxygen consumption, and oxygen diffusion govern the duration for which suitable culture conditions can be maintained [18,19,20]. After adhesion, continuous medium circulation is required to provide nutrients and oxygen. However, fluid flow also generates shear stresses that directly influence cellular behavior. While moderate shear stresses may support physiological activity, excessive mechanical loading can adversely affect cell viability and may promote cell detachment [21,22,23]. Balancing adequate oxygen transport with protection against excessive shear stress requires a robust computational framework capable of characterizing the cellular mechanical environment [24].

Experimental quantification of oxygen depletion and local shear stress in microfluidic devices remains challenging due to the small dimensions involved and the complexity of coupled transport phenomena. Computational modeling therefore provides an attractive alternative for predicting cellular microenvironment conditions and supporting the rational design of microfluidic culture platforms.

Although previous computational studies have investigated oxygen transport and hydrodynamic conditions in microfluidic cell culture systems [18,23,25], generalized predictive correlations for estimating oxygen depletion without repeated numerical simulations [25,26] remain limited. Furthermore, rapid predictive design tools for gas-impermeable architectures remain highly limited compared to conventional gas-permeable PDMS devices [7,17]. Accordingly, this study develops a computational framework to evaluate oxygen availability and wall shear stress simultaneously in SU-8-based microfluidic cell culture chambers. Based on the numerical results, the breakthrough of this work is the proposal of an empirical correlation to predict oxygen concentration as a function of chamber height, providing a rapid predictive tool for device design. The combined oxygen transport and wall shear stress analyses further establish practical design guidelines for selecting chamber geometries and operating conditions that maintain adequate oxygen availability and mechanically favorable conditions for cell adhesion.

## 2. Materials and Methods

### 2.1. Microfluidic Chip Geometry

**Figure 1:**
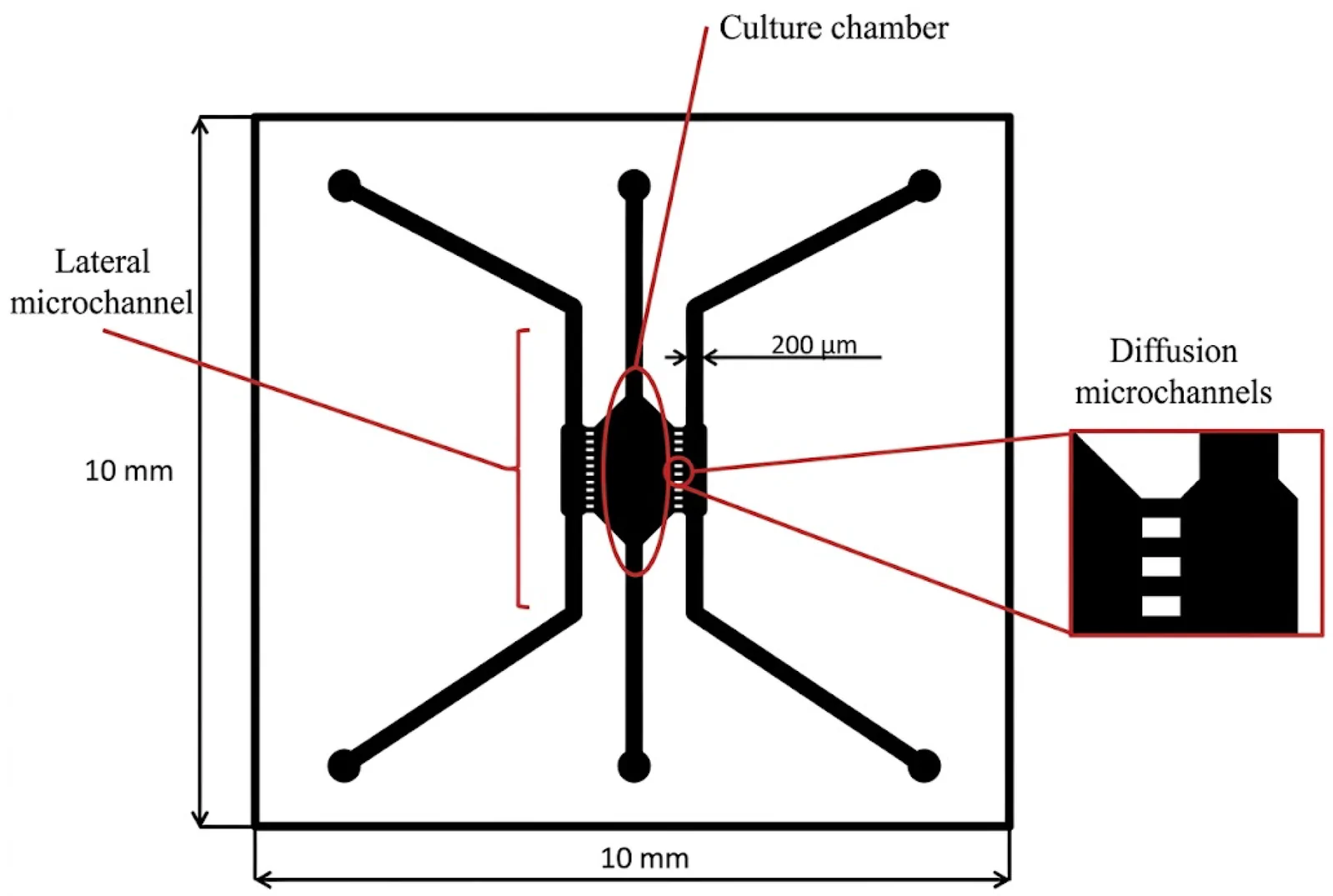
Schematic representation of the proposed microfluidic chip showing the central culture chamber, lateral perfusion microchannels, diffusion channels, and the principal dimensions considered in the computational model.

In this study, we propose a microfluidic chip geometry consisting of a central cell culture chamber connected to two lateral perfusion microchannels through narrow diffusion channels, as commonly used in microfluidic cell culture applications [8,27,28]. A schematic representation of the proposed microfluidic device and its main dimensions is presented in Figure 1. The central chamber, designed as the primary cell culture region, features planar dimensions of 1 mm × 1 mm, while the lateral microchannels are 200 μm wide. The communication channels connecting the lateral microchannels to the culture chamber are 50 μm wide.

The geometry of the microfluidic chip is designed to promote efficient transport of oxygen and nutrients while minimizing the direct exposure of cultured cells to high flow velocities, adhering to general design principles found in microfluidic cell culture systems [6,8]. In this configuration, the culture chamber is supplied indirectly through the lateral microchannels, allowing the establishment of controlled transport conditions within the cell culture region.

To investigate the influence of geometry on oxygen availability and hydrodynamic conditions, in the computational analysis, chamber heights ranging from 150–300 μm in 30 μm intervals were considered. Since the height of the chamber directly affects both the amount of oxygen initially available during the static adhesion phase and the shear stress generated by the wall during subsequent perfusion, this parameter was selected as the primary design variable throughout the study.

The lateral perfusion configuration was adopted to avoid direct exposure of the adhered cells to excessive flow velocities while ensuring adequate transport of oxygen and nutrients to the culture chamber. This design allows the use of practical inlet flow rates while maintaining physiologically relevant shear stress levels within the cell culture region [6,23].

The device material was assumed to be SU-8 photoresist, which is widely used in microfluidic fabrication due to its high mechanical strength, optical transparency, chemical stability, and capability to produce high-aspect-ratio microstructures [14,15,16]. Owing to its negligible gas permeability compared with PDMS, oxygen transport through the device walls was neglected in the present simulations.

### 2.2. Oxygen Transport Modeling

Oxygen transport inside the microfluidic chamber was modeled to evaluate cell viability during the initial adhesion stage prior to the establishment of medium flow. Since the device was assumed to be fabricated from SU-8 photoresist, oxygen transport through the solid boundaries was neglected owing to the negligible gas permeability of the material.

The transport of dissolved oxygen in the culture medium was described using the transient convection–diffusion equation derived from species mass conservation and Fickian diffusion [18,29]:

(1) $$\frac{\partial }{\partial t}\left(\rho c_{i}\right)+\nabla \cdot \left(\rho uc_{i}\right)+\nabla \cdot \left(J_{i}\;\right)=R,$$

where ρ is the fluid density, $c_{i}$ is the oxygen mass fraction, u is the fluid velocity vector, $J_{i}$ denotes the diffusive oxygen flux, and *R* represents the oxygen consumption term associated with cellular metabolism.

The diffusive oxygen flux was described according to Fick’s law:

(2) $$J_{i}=-\rho D_{i}\nabla c_{i},$$

where $D_{i}$ is the diffusion coefficient of oxygen in the culture medium. Because the initial adhesion phase was analyzed under static conditions without medium circulation, the fluid velocity was assumed to be zero (u=0), reducing oxygen transport to a purely diffusion-controlled process.

Oxygen consumption by the adhered cells was represented as a surface metabolic reaction governed by Michaelis–Menten kinetics, which is commonly used to describe oxygen-dependent cellular metabolism in biological transport models [26]:

(3) $$R=-\frac{V_{O_{2}}c_{O_{2}}}{c_{O_{2}}+K_{h}},$$

where $V_{O_{2}}$ is the cellular oxygen consumption rate, $c_{O_{2}}$ is the local oxygen mass fraction, and $K_{h}$ is the Michaelis–Menten constant.

Initially, the culture medium inside the chamber was assumed to contain a spatially uniform oxygen concentration corresponding to equilibrium with atmospheric conditions. No-flux boundary conditions were imposed on all SU-8 walls, while oxygen consumption was applied at the bottom surface of the chamber representing the adhered cell layer.

### 2.3. Flow and Shear Stress Modeling

Following the initial static adhesion phase, medium perfusion was introduced into the microfluidic device to ensure continuous oxygen and nutrient supply to the cultured cells. The resulting hydrodynamic conditions inside the microchamber were numerically analyzed to evaluate the WSS generated under different flow conditions.

The culture medium was assumed to be an incompressible, Newtonian fluid operating under laminar flow conditions due to the small characteristic dimensions and low Reynolds numbers typically encountered in microfluidic systems [30]. Under these assumptions, the flow field was governed by the continuity and Navier–Stokes equations:

(4) ∇·u=0,

(5) $$\rho \left(\frac{\partial u}{\partial t}+u.\nabla u\right)=-\nabla p+\mu \nabla^{2}u,$$

where *p* is the pressure, and μ is the dynamic viscosity of the culture medium. A prescribed flow rate was applied at the inlet boundaries of the lateral perfusion microchannels, while atmospheric pressure conditions were imposed at the outlets. No-slip boundary conditions were applied at all solid walls of the microfluidic device. The WSS acting on the cell culture surface was calculated from the local velocity gradients according to:

(6) $$\tau_{w}=\mu \frac{\partial u}{\partial n}\;\;,$$

where $\tau_{w}$ denotes the WSS and ∂u/∂n represents the velocity gradient normal to the wall surface. Different inlet flow rates and chamber heights were investigated to evaluate their influence on the hydrodynamic conditions within the culture chamber, with particular attention devoted to identifying flow conditions capable of maintaining physiologically compatible shear stress levels during perfusion [23,31,32]. Utilizing rigorous mathematical formulations and parametric analysis to characterize distinct cell culture stages inside microfluidic environments serves as a well-established design strategy [25].

### 2.4. Boundary Conditions and Material Properties

The computational simulations were performed under isothermal conditions, assuming constant fluid properties throughout the microfluidic device. The culture medium was modeled as an incompressible Newtonian fluid, and all material properties used in the simulations are summarized in Table 1.

For the oxygen transport simulations, the initial oxygen mass fraction was assumed to be spatially uniform throughout the culture chamber and equal to the oxygen concentration in fresh culture medium under equilibrium conditions. Since the microfluidic device was assumed to be fabricated from SU-8 photoresist, oxygen transport through the chamber walls was neglected. Consequently, no-flux boundary conditions were imposed at all solid surfaces:

(7) $$n\cdot J_{O_{2}}=0,$$

where n denotes the outward unit normal vector to the wall surface and $J_{O_{2}}$ is the diffusive oxygen flux.

Cellular oxygen consumption was imposed at the bottom surface of the culture chamber, corresponding to the adhered cell layer. Oxygen uptake was described using the Michaelis–Menten formulation presented in Equation (3).

For the hydrodynamic simulations, prescribed volumetric flow rates were imposed at the inlets of the lateral perfusion microchannels, while atmospheric pressure conditions were specified at the outlet boundaries. No-slip boundary conditions were applied at all solid surfaces.

The simulations were repeated for different chamber heights and inlet flow rates in order to investigate their influence on oxygen availability and WSS within the culture chamber.

### 2.5. Numerical Implementation

**Figure 2:**
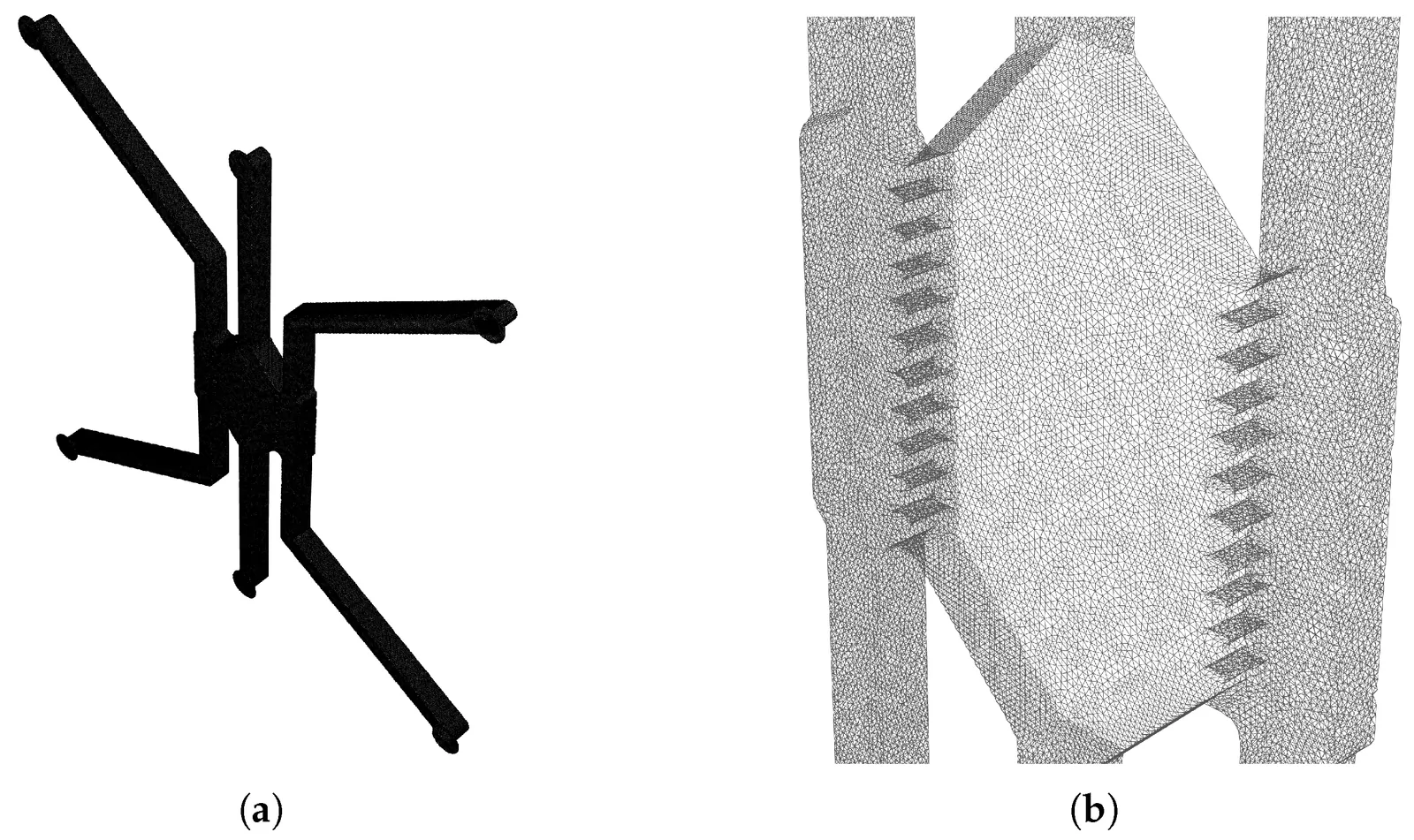
Computational domain and grid generation corresponding to the 300 μm chamber height: (**a**) Global isometric view of the complete multi-channel fluidic device domain; (**b**) Detailed top-down view of the high-density mesh distribution within the central chamber and around the internal microstructures.

The computational domain was discretized into a total of elements ranging from 2.2 million (for the 150 µm chamber height) to 3.2 million (for the 300 µm chamber height) (Figure 2) using a high-density, structured hex-dominant mesh to capture localized velocity gradients near the solid boundaries accurately. The remaining four intermediate chamber heights between 150 µm and 300 µm were systematically adapted within this range of 2.2 to 3.2 million elements. To verify that the numerical solutions were independent of spatial discretization, a mesh sensitivity analysis was performed. The evaluation was carried out on the most geometrically constrained configuration (150 μm chamber height) under the lower cell oxygen consumption rate, which represents the most sensitive regime for capturing subtle diffusion gradients. Three successively refined meshes comprising ∼0.5 million (Coarse), ∼1.8 million (Medium), and ∼2.2 million (Fine) elements were analyzed. The relative variation in the predicted average oxygen concentration was ∼16% when refining the mesh from 0.5 million to 1.8 million elements, which dropped to a negligible ∼1.2% between the 1.8 million and 2.2 million element grids. Furthermore, qualitative and quantitative inspection of the WSS contours demonstrated that the spatial distribution, boundary layer resolution, and peak shear zones achieved full convergence and became invariant beyond the 1.8 million mesh level. Consequently, the spatial resolution corresponding to the 2.2 million element grid was adopted as the independent mesh threshold and consistently utilized for all subsequent parametric variations. To guarantee the mathematical validity and stability of the numerical calculations, mesh metrics were thoroughly evaluated, maintaining a minimum orthogonal quality of 0.366, which safely exceeds standard recommended simulation tolerances.

The fluidic domains were resolved using the steady-state, pressure-based ANSYS Fluent 2021 R2 solver. Given the constant volumetric perfusion rates, a steady laminar regime was modeled. Discretization of momentum and pressure equations utilized a second-order upwind scheme, and convergence was established when the normalized residuals for continuity and velocity components dropped below $10^{-5}$.

For the transient oxygen transport and depletion modeling, the species transport module was enabled to solve the spatial and temporal gas diffusion equations coupled with cell metabolic consumption. Convergence for chemical species tracking was strictly enforced with a normalized residual tolerance below $10^{-6}$. To capture the developing hypoxic front across varying chamber heights while ensuring temporal discretization independence, a second-order implicit time-stepping scheme was employed. A comprehensive temporal sensitivity analysis was conducted on 150 μm chamber height under maximum oxygen consumption using uniform time steps of Δt=5s, Δt=1s, and Δt=0.1s. Refining the time step from 5s to 1s yielded a 6% variation in predicted surface oxygen concentrations, indicating that Δt=5s introduced noticeable temporal truncation errors near the cell boundary. Moreover, time steps exceeding 10s resulted in numerical instability, preventing iterative solver convergence. Conversely, further refining the time step to 0.1s produced negligible deviations in spatiotemporal oxygen profiles while notably increasing the computational time. Consequently, Δt=1s was selected for all subsequent transient simulations.

It should be noted that this computational framework is intended as a predictive, pre-fabrication design tool to guide experimental device geometry optimization. To ensure predictive accuracy in the absence of direct experimental data, the numerical model incorporates experimentally benchmarked kinetic constants and transport properties strictly sourced from validated peer-reviewed literature, solved via standard finite volume formulations in ANSYS Fluent.

## 3. Results and Discussion

### 3.1. Oxygen Depletion Under Static Conditions

**Figure 3:**
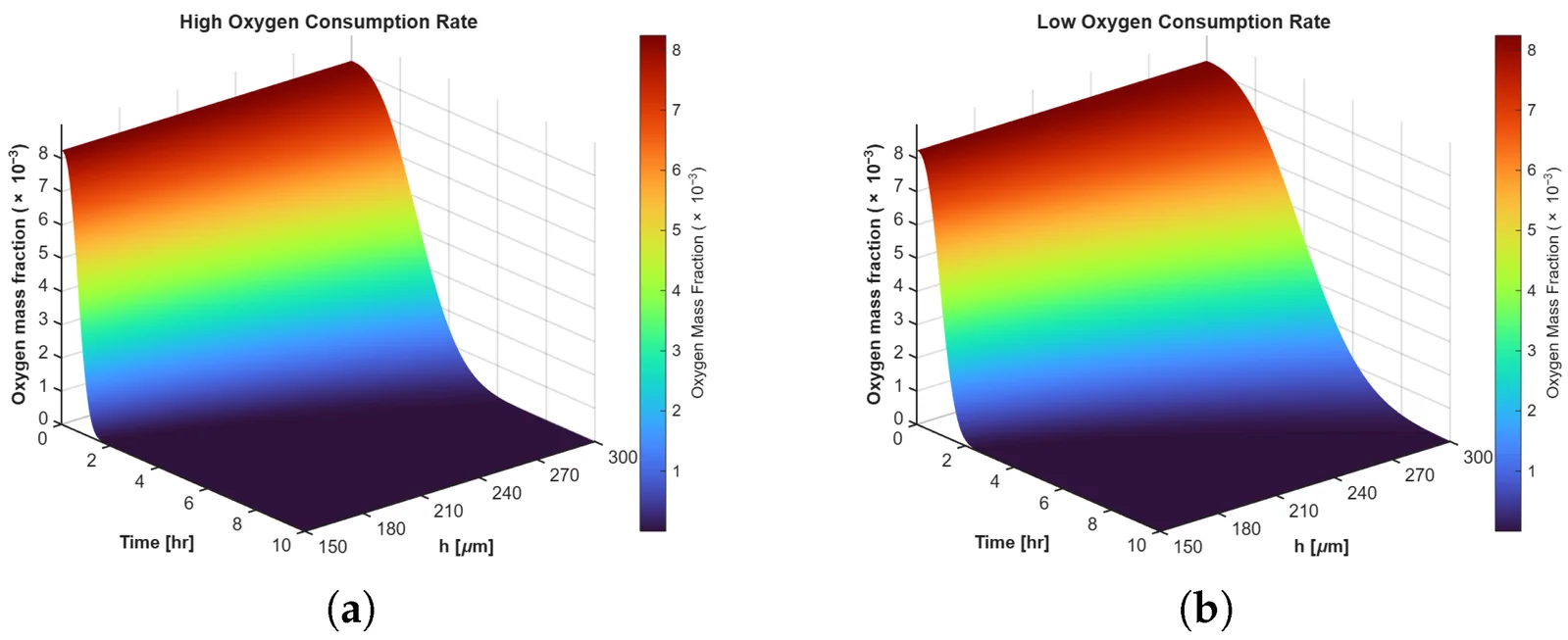
Three-dimensional fitted surfaces representing the temporal evolution of the average oxygen mass fraction at the cell culture surface for chamber heights (h) 150–300 μm in 30 μm intervals under (**a**) high and (**b**) low cellular oxygen consumption rates. The surfaces was generated from the extracted oxygen mass fraction values to provide a continuous representation of oxygen depletion dynamics within the microchamber. Increasing chamber height delays oxygen depletion by increasing the initial oxygen reservoir available within the microchamber. The corresponding dynamic oxygen profiles are provided in Supplementary Video S1.

The temporal evolution of the oxygen concentration within the microfluidic culture chamber was investigated under static conditions prior to the initiation of medium perfusion. The average oxygen mass fraction at the cell culture surface was extracted for different chamber heights (150–300 μm in 30 μm intervals) and for both low and high oxygen consumption rates. A three-dimensional surface was then fitted to these data, as shown in Figure 3. Specifically, the low oxygen consumption rate ($4.4\times 10^{-17}\;\mathrm{mol}\;s^{-1}\;{\mathrm{cell}}^{-1}$) corresponds to human fibroblasts [36], whereas the high oxygen consumption rate ($7.5\times 10^{-16}\;\mathrm{mol}\;s^{-1}\;{\mathrm{cell}}^{-1}$) corresponds to human neurons. A uniform cell seeding density of $8\times 10^{4}\;\mathrm{cells}/{\mathrm{cm}}^{2}$ was assumed for all simulations [37]. Neurons were selected as a high-metabolic-demand benchmark because of their strong dependence on mitochondrial oxidative respiration for energy production [35].

In the absence of external oxygen replenishment, the oxygen concentration progressively decreased as a consequence of cellular metabolic activity. The simulations demonstrate that chamber height has a pronounced influence on oxygen availability within the microchamber. Increasing the chamber height resulted in slower oxygen depletion due to the larger volume of dissolved oxygen initially available inside the chamber.

Cellular metabolic activity also significantly affected the depletion dynamics. As expected, higher oxygen consumption rates produced a more rapid decrease in oxygen concentration, whereas lower consumption rates delayed oxygen depletion. For all simulated configurations, oxygen concentration exhibited a continuous nonlinear decrease with time, with depletion occurring more rapidly as the available oxygen became progressively exhausted.

A comparison between the different chamber heights indicates that increasing the chamber height substantially prolongs oxygen availability during the static adhesion stage. This behavior highlights the importance of chamber geometry in SU-8 microfluidic devices, where oxygen replenishment through the chamber walls is negligible and the initial dissolved oxygen content represents the only oxygen source prior to perfusion.

The simulated transient oxygen depletion profiles under static cell seeding conditions demonstrate a severe drop in localized gas availability within the sealed SU-8 culture chamber. This rapid depletion aligns closely with experimental observations of metabolic consumption kinetics in gas-impermeable microfluidic platforms. Specifically, Ochs et al. demonstrated that highly metabolic cells can exhaust almost all dissolved oxygen within 60 min when confined inside impermeable thermoplastic architectures under static conditions [17]. Because SU-8 exhibits a similarly negligible gas permeability compared to conventional polydimethylsiloxane (PDMS), the reliance on the initial media volume as a finite oxygen reservoir inevitably accelerates the onset of localized hypoxia. This transport limitation is further corroborated by recent studies in PDMS-free microfluidics, which emphasize that dense cell populations rapidly render their local microenvironment overly hypoxic during the critical attachment phase if active perfusion is withheld [38]. Consequently, the non-linear decay curves predicted by our computational framework successfully capture the real-world physical and biological mass transport constraints inherent to rigid, gas-impermeable matrices, qualitatively validating the model’s capacity to establish safe pre-perfusion time windows.

**Figure 4:**
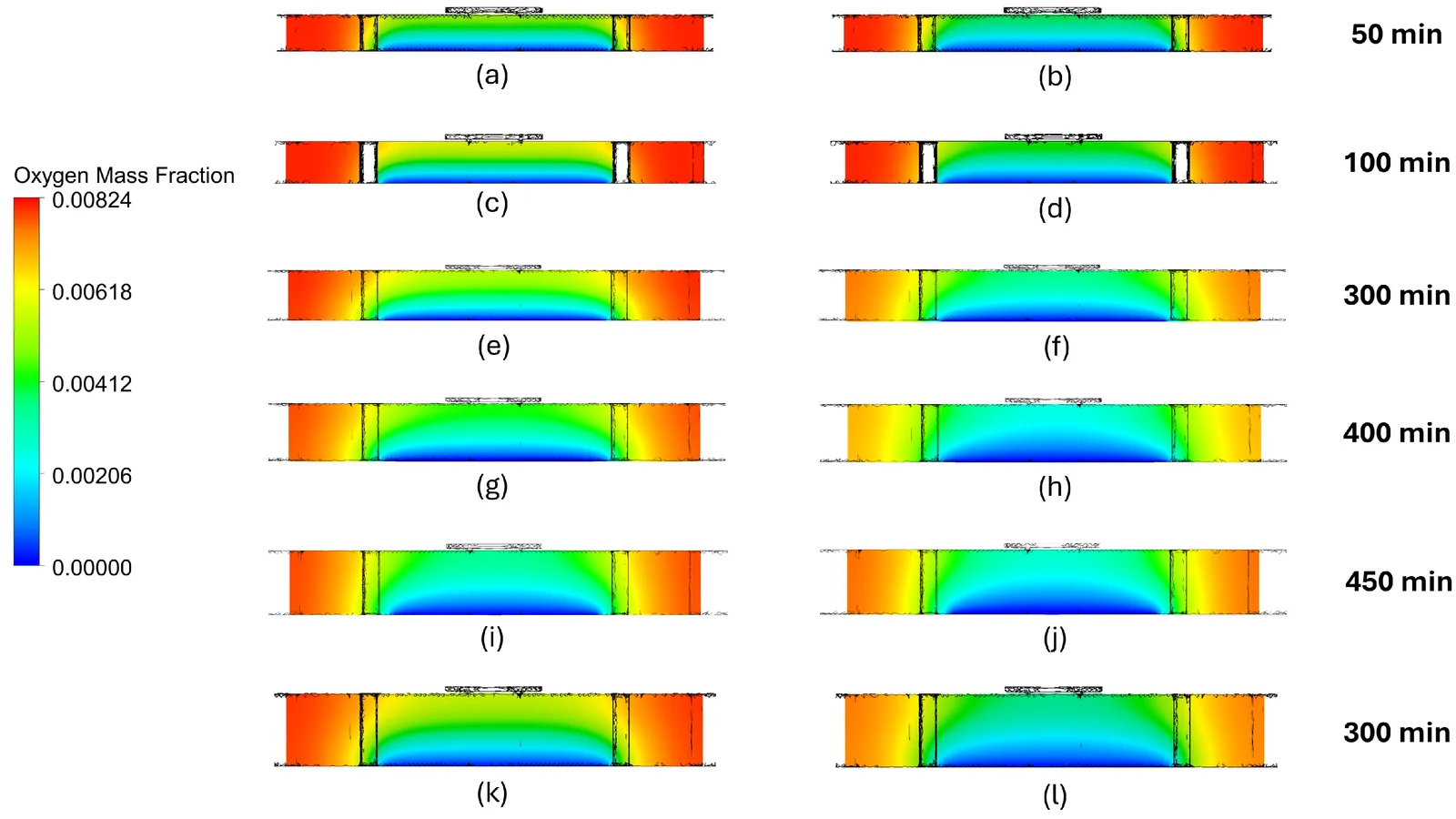
Spatial distribution of oxygen mass fraction in a vertical cross section of the culture chamber at different time instants for low (left column) and high (right column) oxygen consumption rates. Results are shown for chamber heights of 150 μm (**a**,**b**), 180 μm (**c**,**d**), 210 μm (**e**,**f**), 240 μm (**g**,**h**), 270 μm (**i**,**j**), and 300 μm (**k**,**l**). Oxygen depletion initiates near the cell layer at the chamber bottom and progressively propagates toward the upper region of the chamber, which acts as an oxygen reservoir (see Supplementary Video S2).

Figure 4 illustrates the spatial distribution of oxygen mass fraction within the culture chamber at different time instants. Oxygen depletion initially occurs near the cell layer located at the chamber bottom, where metabolic consumption is imposed, leading to the development of concentration gradients along the chamber height. The upper region of the chamber acts as an oxygen reservoir and continuously supplies oxygen to the cells through diffusion. Larger chamber heights provide a greater oxygen reserve and therefore maintain higher oxygen concentrations for longer periods before depletion propagates throughout the chamber volume. This volumetric dependency matches advanced numerical tracking models analyzing temporal cell survival and behavior under hypoxic conditions.

### 3.2. Empirical Correlation for Oxygen Availability

Based on the numerical results, an empirical correlation was derived to predict the temporal evolution of the oxygen concentration within the microchamber as a function of its height:

(8) $$c_{O_{2}}=c_{0}\exp\left[-{\left(\frac{Kt}{h^{2}}\right)}^{n}\right],$$

where $c_{O_{2}}$ denotes the oxygen mass fraction at time *t* in seconds [s], *h* is the chamber height in micrometers [μm], and $c_{0}$ represents the initial oxygen mass fraction, which was set to the standard atmospheric-equilibrium value 0.00824 in all simulations. The remaining parameters, *K* and *n*, are fitting coefficients determined separately for each metabolic condition.

The empirical coefficients *K* and *n* were determined by nonlinear least-squares fitting using the complete dataset comprising chamber heights ranging from 150–300 μm in 30 μm intervals. The fitted coefficients for both high and low oxygen consumption rates are summarized in Table 2. The resulting correlation showed good agreement with the numerical simulations. The goodness of fit was quantified using the coefficient of determination ($R^{2}$) and the root mean square error (RMSE), computed over the complete dataset comprising all chamber heights (150–300 μm in 30 μm intervals) and simulated time points. Both RMSE and *R*^2^ were calculated to provide complementary evaluations of model performance, where *R*^2^ quantifies the proportion of variance explained by the model, while RMSE measures the absolute prediction error in the original measurement units, allowing assessment of both correlation and accuracy. The coefficient of determination was calculated as

(9) $$R^{2}=1-\frac{\sum_{i=1}^{N}{\left(C_{i}-{\hat{C}}_{i}\right)}^{2}}{\sum_{i=1}^{N}{\left(C_{i}-\bar{C}\right)}^{2}},$$

where $C_{i}$ and ${\hat{C}}_{i}$ denote the simulated and predicted oxygen mass fractions, respectively, $\bar{C}$ is the mean simulated oxygen mass fraction, and *N* is the total number of data points. The RMSE was computed as

(10) $$\mathrm{RMSE}=\sqrt{\frac{1}{N}\sum_{i=1}^{N}{\left(C_{i}-{\hat{C}}_{i}\right)}^{2}}.$$

The resulting $R^{2}$ and RMSE values for both metabolic regimes are reported in Table 2, demonstrating good agreement between the empirical correlation and the numerical simulations. The proposed correlation is applicable to chamber heights ranging from 150 to 300 μm, the oxygen consumption rates investigated in this study, and the specific SU-8 microfluidic chamber geometry. Its application beyond these conditions should be undertaken with caution and supported by additional numerical or experimental validation. The appearance of the characteristic length scale $h^{2}$ reflects the dominant role of diffusion in governing oxygen transport within the sealed microchamber.

The proposed relationship provides a simple predictive tool for estimating oxygen availability without performing additional numerical simulations (see Supplementary Video S1). Consequently, it may be used as a practical design guideline for selecting chamber geometries capable of maintaining adequate oxygen levels during the cell adhesion stage.

### 3.3. Numerical Analysis of Fluid Flow and WSS

To evaluate the mechanical microenvironment experienced by the cultured cells, numerical simulations of the fluid flow were performed under two distinct chamber heights (h=150 μm to h=300 μm in 30 μm intervals) at a constant volumetric flow rate of Q=10μL/min. The primary objective was to ensure that the induced WSS remains below the critical physiological threshold for fibroblast cells, widely reported as $\tau_{\mathrm{crit}}\approx$ 0.02 Pa [39], above which cell detachment and altered mechanotransduction signaling occur.

**Figure 5:**
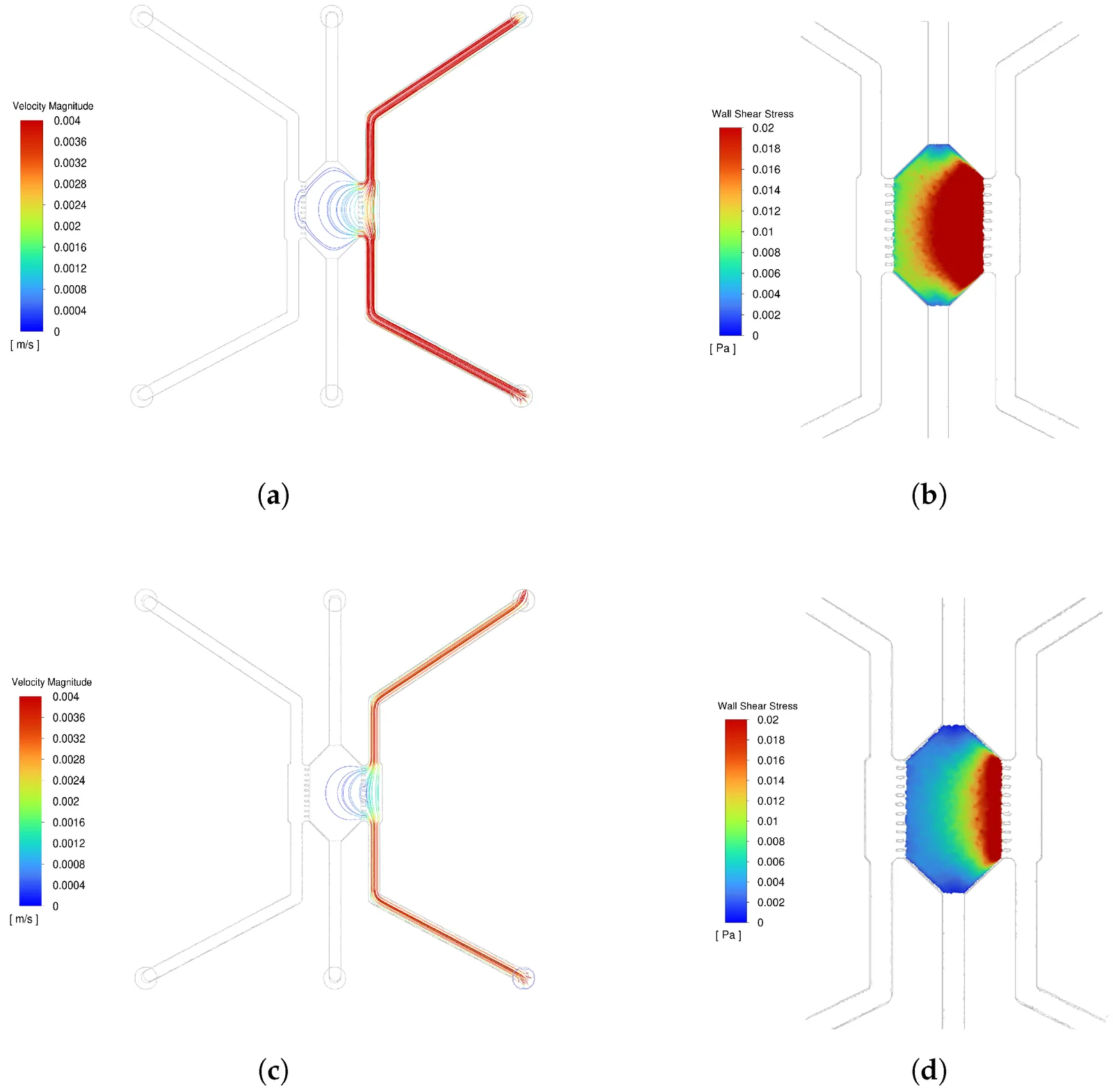
Numerical simulation of fluid flow and WSS distribution at a constant flow rate of 10 μL/min: (**a**) Velocity pathlines (see Supplementary Video S3a) and (**b**) corresponding bottom-WSS contour for the 150 μm high chamber; (**c**) Velocity pathlines (see Supplementary Video S3b) and (**d**) corresponding bottom-WSS contour for the 300 μm high chamber. The red domain in (**b**,**d**) highlights the critical region exceeding the fibroblast physiological threshold ($\tau_{\mathrm{crit}}=0.02\;\mathrm{Pa}$).

Figure 5 presents the velocity pathlines across the microfluidic network alongside the corresponding WSS profiles developed on the cell-attachment wall of the culture chamber.

For the microfluidic chip with h=150 μm (Figure 5a,b), the restricted cross-sectional area significantly intensifies the velocity gradients. This geometric limitation results in a critical and highly unfavorable mechanical regime. As depicted in the WSS contour (Figure 5b), a substantial portion of the chamber bed experiences maximum shear stress values reaching or exceeding the critical threshold of 0.02Pa (represented by the prominent red domain). Operating under these conditions poses a severe risk of disrupting fibroblast adhesion, causing membrane damage, or inducing premature cellular detachment.

Conversely, doubling the channel height to h=300 μm at the same flow rate (Figure 5c,d) drastically mitigates the hydrodynamic forces over the culture zone. The expanded volume effectively dissipates the fluid kinetic energy, yielding a safe and uniform shear profile. The maximum WSS drops well below the 0.02Pa limit, establishing an optimized, low-shear microenvironment conducive to long-term fibroblast proliferation. Consequently, while a flow rate of 10 μL/min is required for convective nutrient delivery, it must be paired with an increased chamber height of h=300 μm to circumvent the cytotoxic, high-shear regimes inherent to the narrower 150 μm geometry.

**Figure 6:**
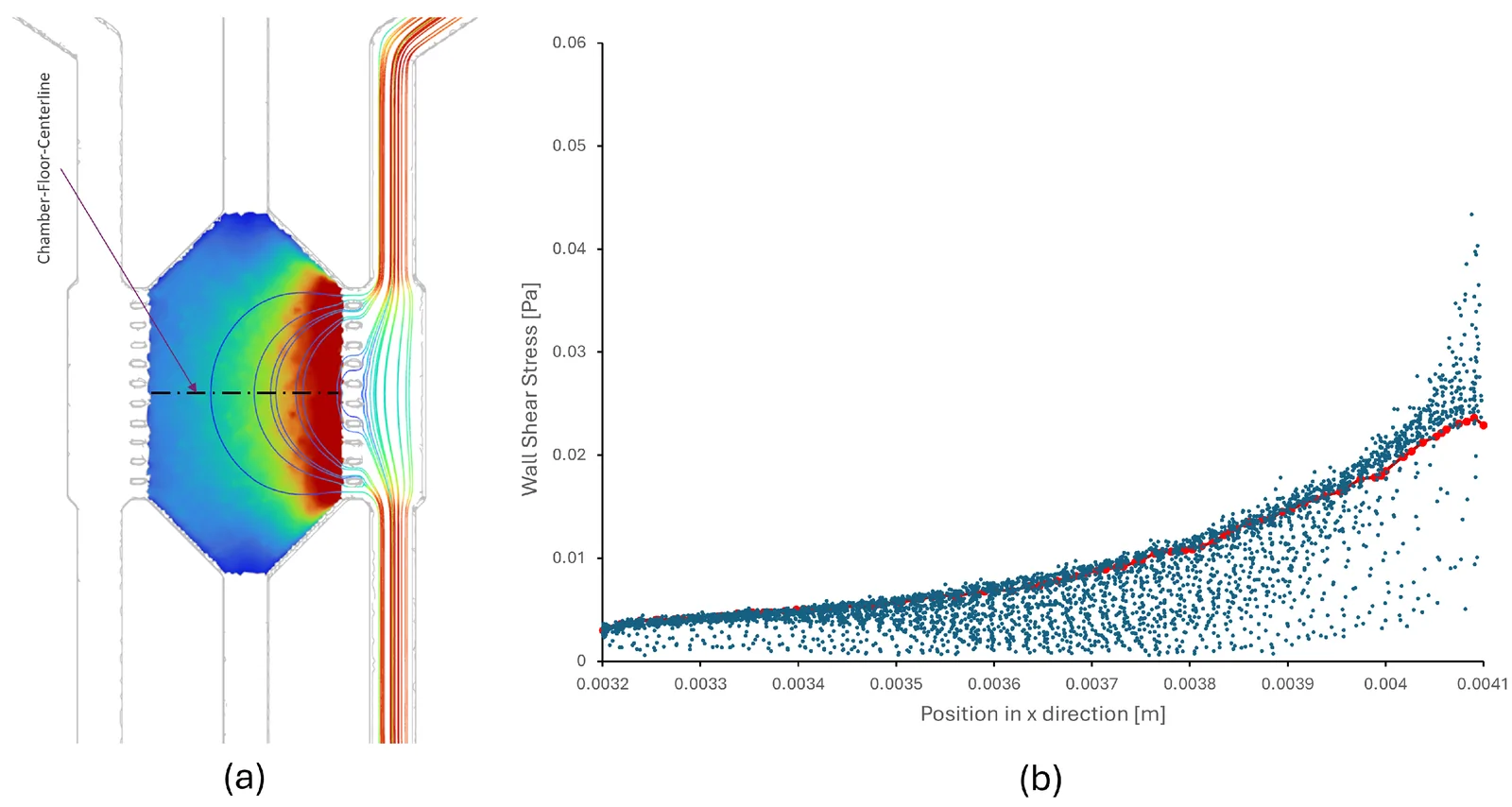
Quantitative analysis of the WSS distribution across the optimized 300 μm chamber floor: (**a**) Spatial definition of the chamber centerline (Chamber-Floor-Centerline) on the cell culture surface, outlined over the WSS distribution results shown in Figure 5b over the WSS contour; (**b**) Localized WSS values as a function of chamber position along the Chamber-Floor-Centerline, comparing the individual cell positions (blue dots) with the central axis profile (red line).

To further provide a quantitative validation of the mechanical environment within this optimized geometry (h=300 μm), the localized WSS was extracted across the cell culture matrix. Figure 6 plots the WSS distribution as a function of the spatial position along the chamber floor, highlighting the discrete data points at the cell positions alongside the continuous profile along the chamber centerline (Chamber-Floor-Centerline) on the cell culture surface.

As numerically demonstrated in the scatter profile, a pronounced spatial heterogeneity in the hydrodynamic forces is observed. Near the channel junctions (x>0.0039m), the fluid velocity magnitudes peak, generating an elevated shear stress zone where values markedly exceed the critical physiological limit of 0.02Pa, reaching up to approximately 0.045Pa. This high-shear boundary zone represents a hostile microenvironment where cell attachment is highly unfavorable due to excessive hydrodynamic drag.

Conversely, as the fluid moves away from the inlet channel and expands into the wider core of the hexagonal culture chamber, a rapid decay in both velocity magnitude and velocity gradients occurs. Consequently, the localized WSS progressively reduces, stabilizing well below the critical threshold (<0.01 Pa) in the central and detached regions of the chamber (x<0.0036m). This quantitative trend rigorously confirms that the spatial zones furthest from the fluidic channels offer the most stable and biocompatible mechanical microenvironment, ensuring optimal conditions for long-term fibroblast anchoring, focal adhesion formation, and undisturbed proliferation.

**Figure 7:**
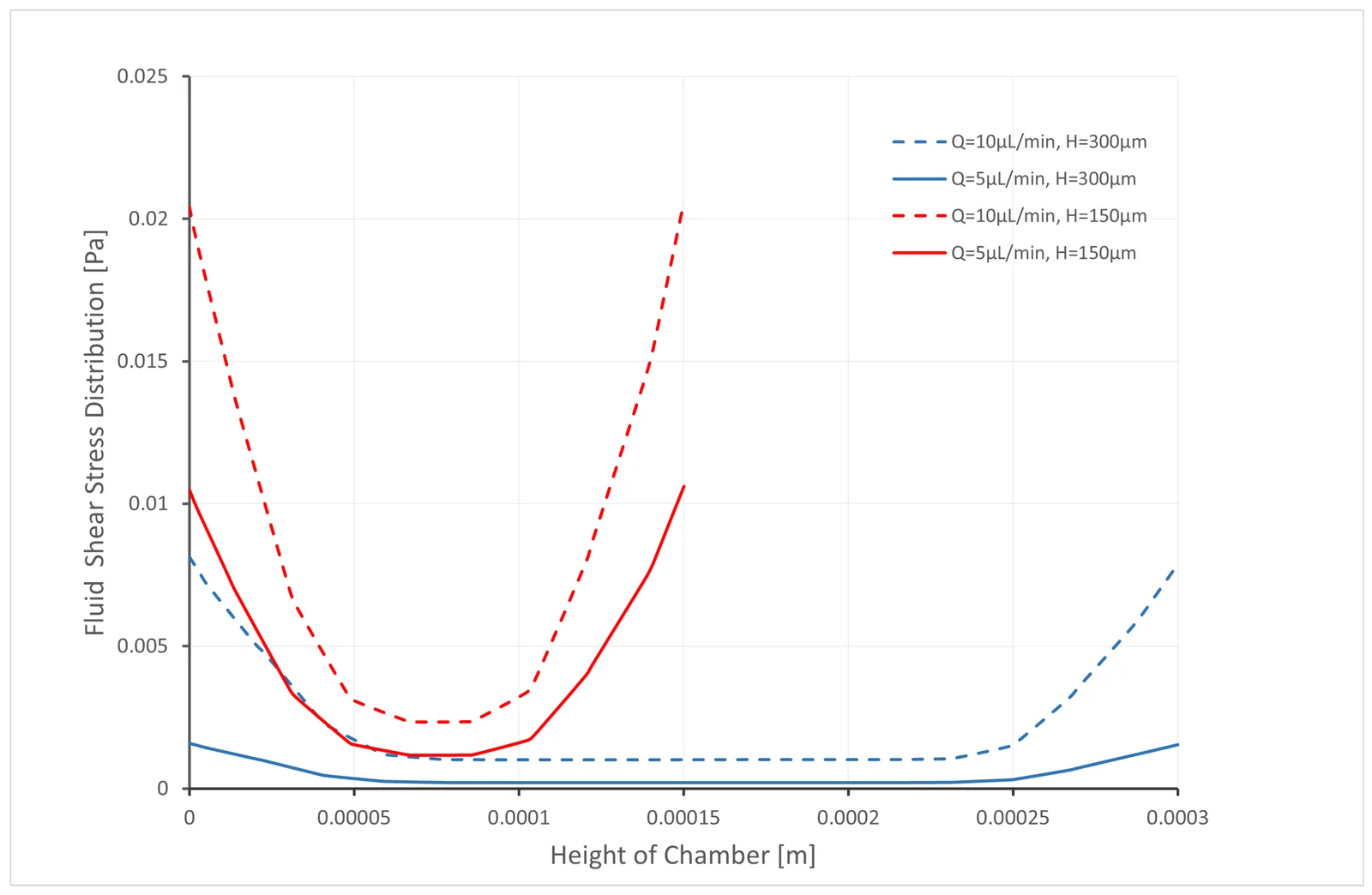
Shear stress distribution profiles plotted across the vertical height axis of the culture chamber, comparing the effects of different chamber heights (h=150 μm vs. 300 μm) and volumetric flow rates (Q=5 μL/min vs. 10 μL/min). The symmetric profiles demonstrate peak shear stresses at the solid microchip boundaries (y=0 and y=h).

To evaluate the hydrodynamics across the chamber height, the fluid shear stress Distribution was analyzed along the vertical axis of the chamber, extending from the bottom surface (y=0) to the chamber ceiling (y=h). As shown in Figure 7, all configurations exhibit a classic symmetric, parabolic profile, where shear stress peaks at the boundaries due to maximum velocity gradients and approaches minimum values at the mid-plane. A comparative analysis highlights the combined influence of chamber geometry and volumetric flow rate on the mechanical forces at the cell-attachment floor (y=0). For the 150 μm geometry at Q=10 μL/min (dashed red curve), the WSS reaches ∼0.021 Pa, which exceeds the safe fibroblast physiological limit ($\tau_{\mathrm{crit}}\approx 0.02$ Pa). While reducing the flow rate to 5 μL/min (solid red curve) lowers the stress to 0.011 Pa, it offers a narrow margin of safety for operational fluctuations. Conversely, doubling the chamber height to 300 μm significantly suppresses the boundary shear. At Q=10 μL/min (dashed blue curve), the maximum WSS stabilizes at ∼0.008 Pa, providing an ideal low-shear window. When the flow rate is decreased to 5 μL/min (solid blue curve) in this larger geometry, the shear stress drops near-negligibly below 0.002 Pa, which may limit convective mass transport and nutrient replenishment. These vertical profiles demonstrate that pairing h=300 μm with Q=10 μL/min ensures an optimal microenvironment, maintaining safe mechanical shear without compromising necessary fluidic throughput.

The computed hydrodynamic profiles and wall shear stress (WSS) thresholds within the microfluidic cell culture domain are highly consistent with experimental cell attachment dynamics reported in literature. Numerically, our framework demonstrates that reducing the chamber height to 150 μm generates elevated local boundary forces that reach or exceed the physiological detachment threshold of 0.021 Pa [39]. To validate these mechanics qualitatively and quantitatively, our findings can be compared to the experimental microfluidic cell adhesion assays conducted by Plouffe et al. [40]. Their work explicitly establishes that stable fibroblast anchoring and high-density cell capture succeed primarily under tightly controlled, low-shear regimes, documenting substantial and efficient fibroblast surface adhesion at an optimal shear stress value of 0.7 dyn cm^−2^ (0.07 Pa). When fluidic drag forces increase beyond this operational envelope, cell-wall anchoring becomes severely compromised. Quantitatively, the proposed optimized 300 μm chamber geometry successfully suppresses the maximum boundary WSS to approximately 0.008 Pa [39], positioning the entire culture bed safely within the stable attachment window validated by their experimental data. Conversely, the high-velocity boundaries near our channel junctions exceed 0.021 Pa and approach their critical operational limit, rigorously confirming that structural height optimization is required to prevent fluid-induced cell detachment. 

## 4. Conclusions

In this study, a comprehensive computational framework was successfully established to model and optimize the coupled transport phenomena and hydrodynamic conditions within an impermeable SU-8-based microfluidic cell culture chip. By systematically evaluating both the static seeding/adhesion phase and the active perfusion stage, this work provides predictive guidelines to achieve an ideal balance between cellular oxygen availability and mechanical safety.

Several key contributions and findings emerge from this study. First, under static conditions representing the initial cell seeding phase, chamber height serves as the governing factor for cell survival time by functioning as a closed oxygen reservoir. Larger chamber heights drastically delay the propagation of hypoxia from the metabolically active cell layer at the lower wall up toward the upper ceiling. To bypass the need for repetitive, resource-intensive numerical simulations, a novel empirical mathematical correlation ($c_{O_{2}}=c_{0}\exp\left[-{\left(Kt/h^{2}\right)}^{n}\right]$) was derived. Incorporating a characteristic diffusion length scale ($h^{2}$), this empirical tool accurately predicts temporal oxygen consumption for the investigated SU-8-based microfluidic chamber geometry, chamber heights between 150 and 300 μm, and the oxygen consumption rates considered in this study. Upon initiating active perfusion at Q=10 μL/min, the hydrodynamic conditions shift significantly. The 150 μm channel height was found to induce a hostile, high-shear boundary regime (≈0.021Pa) that violates the physiological shear threshold for fibroblasts ($\tau_{crit}\approx 0.02\;\mathrm{Pa}$), risking cellular detachment. Conversely, doubling the vertical domain to an optimized height of h=300 μm effectively dissipates fluid kinetic energy. This design choice stabilizes peak boundary shear stresses at a highly biocompatible level (≈0.008Pa) while maintaining sufficient convective throughput to ensure long-term, uninhibited fibroblast anchoring and expansion. Finally, quantitative spatial mapping along the microfluidic chip floor confirmed a distinct spatial heterogeneity, showing that the central core of the hexagonal chamber, farthest from the high-velocity inlet/outlet junctions, yields the most mechanically stable and sheltered zone for focal adhesion formation. Crucially, the predictive capability of this computational framework is validated by established experimental literature. Our non-linear static oxygen depletion profiles show excellent qualitative agreement with the metabolic decay kinetics observed in gas-impermeable chips [17,38]. Furthermore, the simulated hydrodynamic boundary envelopes are supported both qualitatively and quantitatively by microfluidic cell adhesion assays [40], confirming that suppressing the localized wall shear stress beneath their reported operational thresholds prevents fluid-induced fibroblast detachment.

It should be noted that establishing these geometric and hydrodynamic boundaries provides a direct mechanism to guarantee device performance. By keeping localized WSS safely within the biocompatible limits required for stable cell adhesion, as characterized in microfluidic cell isolation assays [40], designers can prevent the cellular detachment and membrane damage that otherwise trigger signal drift in electrochemical biosensors or ruin barrier integrity in organ-on-a-chip systems.

Collectively, the proposed guidelines provide practical design criteria for rigid SU-8-based microfluidic cell culture platforms, including organ-on-a-chip devices and microfluidic biosensors, by enabling the selection of chamber geometries and operating conditions that maintain adequate oxygen availability and physiologically acceptable wall shear stress. These recommendations are applicable to the investigated SU-8-based microfluidic chamber geometry and are based on the physiological wall shear stress thresholds adopted for the investigated cell types.

## Figures and Tables

**Table 1:** Material properties and model parameters used in the computational simulations.

Parameter	Value	Unit	Reference
Fluid density, ρ	1000	$\mathrm{kg}/m^{3}$	[33]
Dynamic viscosity, μ	$1.003\times 10^{-3}$	Pa·s	[33]
Oxygen diffusion coefficient, $D_{O_{2}}$	$2.1\times 10^{-9}$	$m^{2}/s$	[34]
Initial oxygen mass fraction	$8.24\times 10^{-3}$	−	[17]
Michaelis–Menten constant, $K_{h}$	$4.05\times 10^{-3}$	$\mathrm{mol}/m^{3}$	[26]
High oxygen consumption rate, $V_{O_{2}}$	$7.5\times 10^{-16}$	mol/s per cell	[35]
Low oxygen consumption rate, $V_{O_{2}}$	$4.4\times 10^{-17}$	mol/s per cell	[36]

**Table 2:** Fitted parameters of the empirical model describing oxygen mass fraction evolution as a function of time and chamber height under high and low cellular oxygen consumption rates.

Parameter	*K*	*n*	$R^{2}$	RMSE
High Oxygen consumption rate	7.693	2.519	0.95	0.000986
Low Oxygen consumption rate	5.409	2.535	0.96	0.000976

## Data Availability

All data and results are included in the manuscript.

## Supplementary Materials

The following supporting information can be downloaded at: https://www.mdpi.com/article/10.3390/bios16080414/s1, Video S1: Fitted dynamic oxygen profiles representing the temporal evolution of the average oxygen mass fraction at the cell culture surface for chamber heights ranging from 150 to 300 μm under low and high cellular oxygen consumption rates. The curves were generated by fitting the extracted oxygen mass fraction data to provide a continuous representation of the oxygen depletion dynamics within the microchamber. Increasing the chamber height delays oxygen depletion by increasing the initial oxygen reservoir available within the microchamber; Video S2: Spatial distribution of the oxygen mass fraction in a vertical cross-section of the culture chamber over time for low (left column) and high (right column) cellular oxygen consumption rates. Results are shown for chamber heights of 150 μm (a,b), 180 μm (c,d), 210 μm (e,f), 240 μm (g,h), 270 μm (i,j), and 300 μm (k,l). Oxygen depletion originates near the cell layer at the chamber bottom and progressively propagates toward the upper region of the chamber, which serves as an oxygen reservoir; Video S3a: Numerical simulation of fluid flow represented by velocity pathlines at a constant flow rate of 10 μL/min for the 150 μm-high chamber; Video S3b: Numerical simulation of fluid flow represented by velocity pathlines at a constant flow rate of 10 μL/min for the 300 μm-high chamber.
